# Prospect of Gum Arabic–Cocoliposome Matrix to Encapsulate Curcumin for Oral Administration

**DOI:** 10.3390/polym16070944

**Published:** 2024-03-29

**Authors:** Dwi Hudiyanti, Muhammad Fuad Al Khafiz, Khairul Anam, Parsaoran Siahaan, Linda Suyati, Sunarsih Sunarsih, Sherllyn Meida Christa

**Affiliations:** 1 Department of Chemistry, Faculty of Science and Mathematics, Diponegoro University, Prof. Jacob Rais Street, Semarang 50275, Central Java, Indonesia; k.anam@live.undip.ac.id (K.A.); siahaan.parsaoran@live.undip.ac.id (P.S.); linda_suyati@live.undip.ac.id (L.S.); 2 Postgraduate Chemistry Program, Faculty of Science and Mathematics, Diponegoro University, Prof. Jacob Rais Street, Semarang 50275, Central Java, Indonesia; queenfoe@gmail.com; 3 Department of Mathematics, Faculty of Science and Mathematics, Diponegoro University, Prof. Jacob Rais Street, Semarang 50275, Central Java, Indonesia; sunarsih@lecturer.undip.ac.id; 4 Chemistry Program, Faculty of Science and Mathematics, Diponegoro University, Prof. Jacob Rais Street, Semarang 50275, Central Java, Indonesia; meidachrista@gmail.com

**Keywords:** antioxidant activity, encapsulation efficiency, loading capacity, release rate, simulated gastric fluid, simulated intestinal fluid

## Abstract

Curcumin is an antioxidant that can effectively eliminate free radicals. However, as its oral bioavailability is low, an effective delivery method is required. Phospholipid-based liposomes can encapsulate lipophilic drugs, such as curcumin, while liposome, cholesterol, and gum Arabic (GA) can enhance the internal and external stability of drug membranes. This present study used concentrations of cholesterol (C_chol_) and GA (C_GA_), ranging from 0 to 10, 20, 30, and 40% as well as 0 to 5, 10, 15, 20, 30, and 40%, respectively, to encapsulate curcumin in a GA–cocoliposome (CCL/GA) matrix and test its efficacy in simulated intestinal fluid (SIF) and simulated gastric fluid (SGF). The absence of new characteristic peaks in the Fourier transform infrared (FTIR) spectra results indicate the presence of non-covalent interactions in the CCL/GA encapsulation. Furthermore, increasing the C_chol_ decreased the encapsulation efficiency (EE), loading capacity (LC), and antioxidant activity (IR) of the CCL/GA encapsulation but increased its release rate (RR). Conversely, increasing C_GA_ increased its EE and IR but decreased its LC and RR. The two conditions applied confirmed this. Liposomal curcumin had the highest IR in SIF (84.081%) and the highest RR in SGF (0.657 ppm/day). Furthermore, liposomes loaded with 10% C_chol_ and 20% C_GA_ performed best in SIF, while those loaded with 10% C_chol_ and 30% C_GA_ performed best in SGF. Lastly, the CCL/GA performed better in SIF than SGF.

## 1. Introduction

Curcumin, a compound that is present in turmeric (*Curcuma longa* L.), possesses anti-inflammatory [1,2], anticancer [2], antibacterial [3], and antioxidant [2] properties. It is a significant antioxidant as it can directly capture and transform free radicals into more stable or non-radical molecules [4]. However, there are limitations of curcumin that diminish its efficacy, namely, (i) its varying solubility, for instance, low solubility (7.8 µg/mL) in water [5], moderate solubility (0.0004 mg/mL) in physiologic pH [6], and excellent solubility (10 mg/mL) in organic solvents, such as 96% ethanol [7]; (ii) the effect of pH on its degradation, for instance, its half-life is 1–9 min at pH 7.2–8.0 (alkaline) and 100–200 min at pH 3.0–6.5 (acidic) [8]; (iii) its low in vivo absorption; (iv) its limited in vivo bioavailability; and (v) its significant metabolism and excretion by the body [9,10]. As such, multiple studies have thoroughly examined overcoming issues in curcumin distribution, primarily by developing drug delivery systems (DDSs) to deliver curcumin to specific in vivo sites [9,11]. Various types of DDSs, such as micelles [12], emulsions [13], solid lipid nanoparticles [14,15,16], nanoparticles [10,17,18], and natural polymer-coated liposomes, such as gum Arabic (GA) [19,20], have been examined as potential curcumin encapsulation.

Multiple studies have approved the use of liposome-based DDSs to administer drugs in vivo [11,21]. Liposomes are hollow spherical vesicles with an aqueous compartment surrounded by multiple layers of phospholipids. The structure of a liposome makes it an ideal DDS as its aqueous compartment entraps hydrophilic or polar drugs while its bilayers entrap lipophilic or non-polar drugs. It is also a non-toxic, biodegradable, and biocompatible DDS that yields high levels of in vivo bioavailability. Furthermore, as liposomes can be engineered at a nanoscale to achieve targeted drug delivery, they can, therefore, be used to enhance the in vivo bioavailability, stability, and solubility of curcumin [6,22].

The components in a DDS must interact synergistically to maintain and enhance drug delivery. Phospholipids are the ideal primary component for liposomes as they can spontaneously create a self-assembled structure in an aqueous solution due to their amphiphilic nature. However, liposomes that solely contain phospholipids tend to leak and release their payload before reaching the target site. Cholesterol can be used to decrease leaks by sandwiching it between the phospholipid layers of the liposome’s membrane, which would affect how the phospholipids are arranged and increase the regularity and rigidity of the membrane, thereby stabilising the permeability of the liposome [23,24]. Apart from cholesterol, a polymer matrix, such as GA, can also be used to coat liposomes and decrease leakage. As GA is a biopolymer, it has excellent water solubility as well as dynamic and high surface activity, which enables it to not only readily interact with the outer surfaces of liposomes [25] but also to create a thin protective layer that increases its stability without increasing the internal density [26].

A comprehensive grasp of all pertinent elements is necessary when developing a liposome-based DDS with which to effectively address drug delivery challenges. One of the many obstacles that liposomes encounter while traversing the gastrointestinal tract is the significant difference in pH. More specifically, the pH of the stomach is 1.5–2.0 [27] while that of the intestine is 6.5–7.5. As such, multiple studies have used solutions that mimic the pH of the intestine (simulated intestinal fluid; SIF) and stomach (simulated gastric fluid; SGF) to examine the influence of changes in pH on liposomes composed of commercially available phospholipids [28,29]. This present study used cocoliposomes derived from coconut phospholipids (CocoPLs), cholesterol [28], and GA [19] to produce multiple encapsulation matrices. At certain cholesterol concentrations (C_chol_), the cocoliposome/cholesterol matrix yielded a low encapsulation efficiency (EE), antioxidant activity (IR) of curcumin, and loading capacity (LC). Meanwhile, the GA/cholesterol matrix yielded high EE and curcumin IR but low LC. Therefore, the cocoliposome–GA/cholesterol matrix was integrated to determine if it would produce disparate or analogous outcomes. This study investigated the EE of a cocoliposome–GA/cholesterol-based curcumin DDS in two distinct oral delivery routes, namely, SIF (slightly alkaline) and SGF (acidic). Although the behaviour of the proposed DDS varied in the two delivery routes, it was a suitable curcumin DDS. Several instruments were used to support the findings of this present study, including a UV-Vis spectrophotometer, a Fourier transform infrared spectrophotometer, and a nanoparticle analyser.

## 2. Materials and Methods

### 2.1. Preparation of 0.1 M of Phosphate-Buffered Saline (PBS) Solution

Solution A (0.1 M) was prepared by dissolving 1.98 g of Na_2_HPO_4_·2H_2_O p.a. (Merck KGaA, Darmstadt, Germany) in 500 mL of demineralised water (Brataco, Semarang, Indonesia), while Solution B (0.1 M) was prepared by dissolving 1.56 g of NaH_2_PO_4_·2H_2_O p.a. (Merck KGaA, Darmstadt, Germany) in 500 mL of demineralised water (Brataco, Semarang, Indonesia). Solutions A (80.2 mL) and B (19.8 mL) were then homogenised before the pH was adjusted to 7.4 using diluted 0.1 M hydrochloric acid (HCl) p.a. or 0.1 M sodium hydroxide (NaOH) p.a.

### 2.2. Preparation of Simulated Intestinal Fluid (SIF) Solution

Solution C (0.05 M) was prepared by dissolving 7.5 g of Na_2_HPO_4_·2H_2_O p.a. (Merck KGaA, Darmstadt, Germany) in 500 mL of demineralised water (Brataco, Semarang, Indonesia), while Solution D (0.05 M) was prepared by dissolving 3.9 g of NaH_2_PO_4_·2H_2_O p.a. (Merck KGaA, Darmstadt, Germany) in 500 mL of demineralised water (Brataco, Semarang, Indonesia). The SIF was prepared by homogenising Solutions C (40.5 mL) and D (9.5 mL) and then diluting the mixture to 100 mL before adjusting the pH to 7.4 using diluted 0.1 M HCl p.a. or 0.1 M NaOH p.a.

### 2.3. Preparation of Simulated Gastric Fluid (SGF) Solution

Two grams (2 g) of sodium chloride (NaCl; HiMedia, Pennsylvania, PA, USA) p.a. was dissolved in 800 mL of demineralised water (Brataco, Semarang, Indonesia) before 4.5 mL of 37% (*w*/*w*) HCl (Merck KGaA, Darmstadt, Germany) p.a. was added drop-wise. The SGF was prepared by re-adding demineralised water (Brataco, Semarang, Indonesia) to a final volume of 1 L and then adjusting the pH to 1.2 using diluted 0.1 M HCl p.a. or 0.1 M NaOH p.a.

### 2.4. Encapsulation of Curcumin in Cocoliposomes with Gum Arabic Matrix

The CocoPLs were extracted in-house using the method outlined by Hudiyanti et al. (2018) [30,31]. In brief, the CocoPLs were extracted from ripe coconut meat using a chloroform (Merck KgaA, Darmstadt, Germany) p.a. and methanol (Merck KgaA, Darmstadt, Germany) p.a. concoction. It was then partitioned using technical n-hexane (Kimia Kalijaga, Demak, Indonesia) and technical 87% (*v*/*v*) ethanol (Kimia Kalijaga, Demak, Indonesia). Table 1 provides the formulations of curcumin encapsulated within cocoliposomes (CCLs), while Table 2 provides the formulations of CCL with GA matrix coating (CCL/GA). Varying concentrations of GA (C_GA_) (0%, 5%, 10%, 20%, 30%, and 40%) were added to each CCL formulation to produce the CCL/GA formulations. The CCL formulations in Table 1 comprised cholesterol p.a. (94%) and curcumin p.a. (≥75%), both from Sigma-Aldrich (Darmstadt, Germany).

The CCL was prepared by dissolving CocoPLs, cholesterol p.a. and curcumin p.a. in a 9:1 (*v*/*v*) ratio of chloroform p.a. (Merck KgaA, Darmstadt, Germany) and methanol p.a. (Merck KgaA, Darmstadt, Germany) solvent. The total volume of the final solution was 100 mL. Ten millilitres (10 mL) of the solution was placed in a test tube and flowed with nitrogen gas (N_2_) until a thin film remained at the bottom of the tube. Ten millilitres (10 mL) of the SIF was then added into the test tube sans agitation before undergoing the freeze–thaw cycle [19,28]. The freeze–thaw procedure involved three sequential steps: (i) heating the solution to 45 °C (thaw), (ii) cooling it to 4 °C (freeze), and then (iii) homogenising with a vortex mixer. The cycle was repeated until the thin film at the bottom of the test tube containing the SIF had completely dissolved. GA was then added to the CCL and homogenised using a vortex mixer until it had completely dissolved. Sonication (40 kHz, 27 °C, 30 min) was then performed to produce the CCL/GA dispersions. These steps were repeated using all the CCL/GA formulations in the SGF and SIF. Next, 1 mL of the CCL/GA dispersion was combined with 5 mL of 96% (*v*/*v*) ethanol p.a. (Merck KgaA, Darmstadt, Germany) and centrifuged at 3461× *g* for 40 min to segregate the upper liquid layer (supernatant containing unencapsulated curcumin) from the lower solid layer (residue containing CCL/GA). The supernatant can be analysed to determine the EE and the LC, while the residue can be analysed to determine the IR and release rate (RR) or stored at −18 °C. The sink temperature of each CCL/GA dispersion was maintained at 30 °C prior to analysis.

### 2.5. Determination of the Functional Groups

The functional groups present in each CCL/GA composition were determined (Table 1 and Table 2) by conducting three scans using a PerkinElmer^®^ Frontier™ Fourier transform infrared (FTIR) spectrophotometer (Waltham, MA, USA) at a spectral range of 5500–435 cm^−1^ and a spectral resolution of 4.0 cm^−1^ to determine if any interactions that alter the chemical structure during encapsulation had occurred. The analysis primarily examined the GA and CCL/GA, as extant studies have already examined the other components, namely, CocoPLs, cholesterol p.a., curcumin p.a. and CCL [28].

### 2.6. Determination of the Encapsulation Efficiency (EE) of the Curcumin

Equation (1) was used to determine the EE of the curcumin p.a. using data on the initial curcumin p.a. concentration (C_0_ curcumin, 10 ppm) and the unencapsulated curcumin p.a. concentration (C_t_, in ppm) present in the centrifuged supernatant. The C_t_ can be determined by quantifying the absorbance of the supernatant at a wavelength (λ_max_) = 426 nm using a Shimadzu^®^ UV-1280 multipurpose ultraviolet–visible (UV-Vis) spectrophotometer (Kyoto, Japan) [19,32].

(1) $$EE=\left[1-\left(\frac{C_{t}\;curcumin}{C_{0}\;curcumin}\right)\right]\times 100\%$$

### 2.7. Determination of the Loading Capacity (LC) of CocoPLs and GA Carriers

Similar to the method of determining the EE of the curcumin p.a., the LC of the CocoPLs and GA also required C_t_ data, which involved quantifying the absorbance of the supernatant at λ_max_ = 426 nm via the UV-Vis specifications in Section 2.6. The obtained C_t_ was inserted into Equation (2) to calculate the LC of the CocoPLs [19], while Equation (3) was used to calculate the LC of the GA [33].

(2) $$LC\;CocoPLs=\frac{\left(C_{0}\;curcumin-C_{t}\;curcumin\right)\times mL\;CCL/GA\;dispersion}{g_{CocoPLs}}$$

(3) $$LC\;GA=\frac{\left(C_{0}\;curcumin-C_{t}\;curcumin\right)\times mL\;CCL/GA\;dispersion}{g_{GA}}$$

The CCL/GA dispersion (mL) indicates the volume of the dispersion used throughout the analysis (1 mL), while g_CocoPLs_ indicates the mass of the CocoPLs used in the formulation (125 mg), and g_GA_ indicates the mass of the GA used in the formulation (Table 1 and Table 2).

### 2.8. Determination of the Release Rate (RR) of Curcumin

Using the UV-Vis specifications in Section 2.6, the RR was determined by measuring the concentration of curcumin p.a. released by the CCL/GA during the observation period (12 days). Firstly, 1 mL of the CCL/GA dispersion was dispersed into 5 mL of the buffer solution (SGF or SIF) and then incubated at 4 °C and observed. Prior to the analysis, the CCL/GA dispersion was ultrasonically homogenised for 5 min to ensure homogeneity and then centrifuged at 4500 rpm for 15 min. The amount of curcumin p.a. released by the CCL/GA dispersion was determined by measuring the absorbance of the upper layer collected post-centrifugation at λ_max_ = 426 nm.

### 2.9. Determination of the Particle Size (PS), Polydispersity Index (PDI), and Zeta Potential (ζ-Potential) of the Liposomes

A Horiba Scientific^®^ nanoPartica SZ-100V2 Series nanoparticle analyser (Kyoto, Japan) was used to perform dynamic light scattering (DLS), at a scattering angle of 173° and a holder temperature of 25 °C, and determine the PS (z-average diameter), polydispersity index (PDI), and zeta potential (ζ-potential) of the liposomes in the SIF. The PS and PDI indicate the size and distribution of the particles within the liposomes, while the ζ-potential reveals the surface charges of the liposomes.

### 2.10. Determination of the Antioxidant Activity (IR) of Curcumin

Th antioxidant activity of the curcumin p.a. was determined by measuring the inhibition rate (IR) of 1-diphenyl-2-picrylhydrazyl (DPPH; ≥90%; Sigma-Aldrich) using the DPPH free radical scavenging assay method [32,34,35]. A total of 1 mL of the CCL/GA dispersion was mixed with 3 mL of the DPPH (40 µg/mL) and incubated in the dark at room temperature for 30 min. The absorbance data obtained using the UV-Vis specifications in Section 2.6 at λ_max_ = 515 nm were input into Equation (4) to calculate the IR.

(4) $$IR=\left[\frac{A_{0}-A_{1}}{A_{0}}\right]\times 100\%$$

A_0_ indicates the absorbance of the DPPH, while A_1_ indicates the absorbance of the DPPH when mixed with the CCL/GA dispersion.

### 2.11. Statistical Analyses

The data were collected in triplicate and evaluated using one-way analysis of variance (ANOVA). The significance level was set at *p* < 0.05. The results were expressed as the mean ± standard deviation and presented in graphical format.

## 3. Results and Discussion

DDSs offer formulations that can be arranged according to the nature of the drug being delivered and its intended use. They can also regulate the dosage of the drug to be delivered to the target site, thereby ensuring that the dosage is not excessive and optimal for the desired therapeutic effects. It is essential to evaluate various factors, such as the nature of the drug being delivered, the nature of the drug carrier, the composition of the formulation [36,37], and the structural integrity of the drug carrier [38], to develop an effective DDS. Curcumin, which is a lipophilic drug, has poor water solubility [5] and suffers from absorption-related issues. Therefore, its delivery method must address these issues. The present study proposed a modified DDS formulation that uses liposomes, comprising CocoPLs, cholesterol, and GA polymers, to effectively encapsulate curcumin. The modifications observed in the formulations of CCL/GA, the EE of curcumin, the LC of the CocoPLs, the LC of the GA, the IR of the curcumin, and the RR of the curcumin indicate that the endeavour was successful. The use of liposomes to encapsulate drugs offers various benefits, such as sustained drug release, specifically targeting colitis tissues by enhancing epithelial permeation and retention (eEPR), and high stability in gastric fluid due to its pseudo-pH-sensitivity properties [39,40,41]. As this present study aimed to investigate the effect of liposome-encapsulated curcumin on the gastrointestinal tract, its performance in two different conditions, namely, in the intestine, where the drug was dissolved in SIF at pH = 7.4, and in the stomach, where it was dissolved in SGF at pH = 1.2, was examined. Specific data, particularly, the mean and the optimum values of each parameter in the two different conditions, are presented in graphical format.

### 3.1. Functional Group Analyses

**Figure 1:**
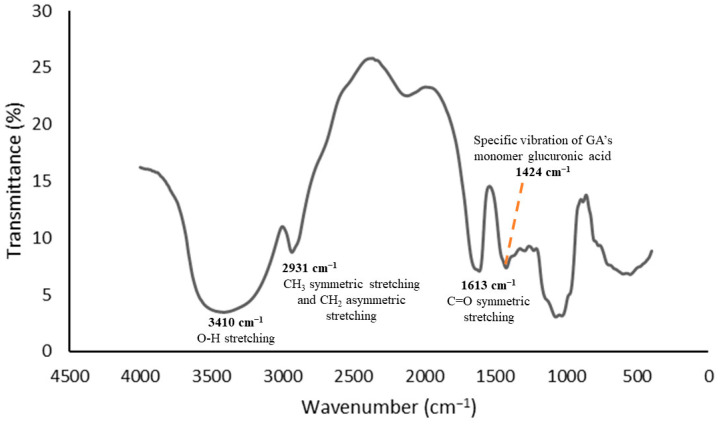
FTIR spectra of the GA.

**Figure 2:**
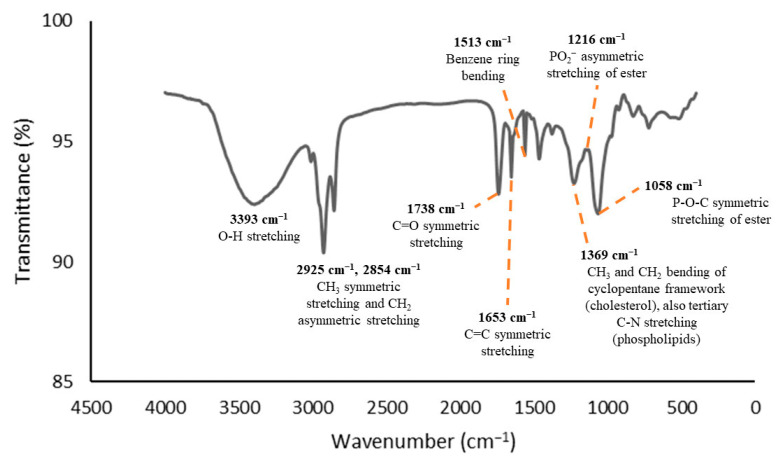
FTIR spectra of the CCL [28].

**Figure 3:**
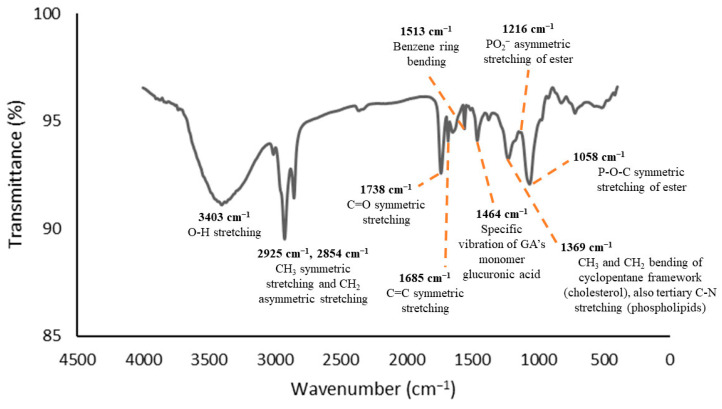
FTIR spectra of the CCL/GA.

The functional group analyses were conducted using FTIR spectroscopy as it provides insights into the interactions between the functional groups of the encapsulated components and the encapsulating components throughout the encapsulation process. The CCL/GA matrix, which served as the curcumin DDS, was designed to prevent any interactions within itself that could alter the chemical structure of the curcumin. The primary purpose of using the DDS was to enhance curcumin transport and release at intended sites in vivo. Therefore, the infrared absorption spectra of the CCL/GA should not exhibit new characteristic peaks that differ from the infrared absorption spectrum of its components. Figure 1, Figure 2 and Figure 3 display the FTIR spectra of the GA, CCL, and CCL/GA, respectively. The present study only examined these three FTIR spectra, as extant studies have already examined the FTIR spectra of CocoPLs, cholesterol, curcumin, and CCL and their corresponding analysis [28].

Figure 1 depicts the characteristic peaks of the GA, which are located at wave numbers (ṽ) = 3410 cm^−1^ (O-H stretching vibration), 2931 cm^−1^ (CH_3_ symmetric stretching and CH_2_ asymmetric stretching vibrations, which indicate the presence of sugars, galactose, arabinose, and rhamnose), 1613 cm^−1^ (C=O symmetric stretching vibration), and 1424 cm^−1^ (specific vibration of the glucuronic acid monomer of GA).

Figure 2 depicts the characteristic peaks of CCL, which are located at ṽ = 3393 cm^−1^ (O-H stretching vibration), 2925 cm^−1^ and 2854 cm^−1^ (CH_3_ symmetric and CH_2_ asymmetric stretching vibrations), 1738 cm^−1^ (C=O symmetric stretching vibration), 1653 cm^−1^ (C=C symmetric stretching vibration), 1513 cm^−1^ (benzene ring bending vibration), 1369 cm^−1^ (CH_3_ and CH_2_ bending vibration of the cyclopentane framework of the cholesterol as well as the tertiary C-N stretching vibration of the phospholipids), 1216 cm^−1^ (PO_2_^−^ asymmetric stretching vibration of the ester), and 1058 cm^−1^ (P-O-C asymmetric stretching vibration of the ester).

The characteristic peaks of CCL/GA can be observed in Figure 3. These peaks are located at ṽ = 3403 cm^−1^ (O-H stretching vibration), 2925 cm^−1^ and 2854 cm^−1^ (CH_3_ symmetric and CH_2_ asymmetric stretching vibrations), 1738 cm^−1^ (C=O symmetric stretching vibration), 1685 cm^−1^ (C=C symmetric stretching vibration), 1513 cm^−1^ (benzene ring bending vibration), 1464 cm^−1^ (specific vibration of the glucuronic acid monomer of the GA), 1369 cm^−1^ (CH_3_ and CH_2_ bending vibration of the cyclopentane framework of the cholesterol as well as the tertiary C-N stretching vibration of the phospholipids), 1216 cm^−1^ (PO_2_^−^ asymmetric stretching vibration of the ester), and ~1058 cm^−1^ (P-O-C asymmetric stretching vibration of the ester). Some of the peaks did shift slightly when the GA matrix was added to the CCL dispersion. For instance, the specific vibration peak of the glucuronic acid monomer of the GA shifted from 1424 to 1464 cm^−1^, while the peak for the C=C symmetric stretching vibration shifts shifted 1653 to 1685 cm^−1^. The increase in ṽ occurred due to interactions between the polymer and liposomes. Nevertheless, more studies, such as computational studies, are required to validate the presence of this interaction.

The spectra of the CocoPLs [28], cholesterol [28], curcumin [28], GA, and CCL [28] indicate that the characteristic peaks of each component were identified and observed in the FTIR spectra of the CCL/GA, sans new characteristic peaks. Therefore, the interactions that occurred while encapsulating the curcumin in the CCL/GA matrix were non-covalent and did not alter the chemical structure.

### 3.2. Encapsulation Efficiency (EE) of Curcumin

**Figure 4:**
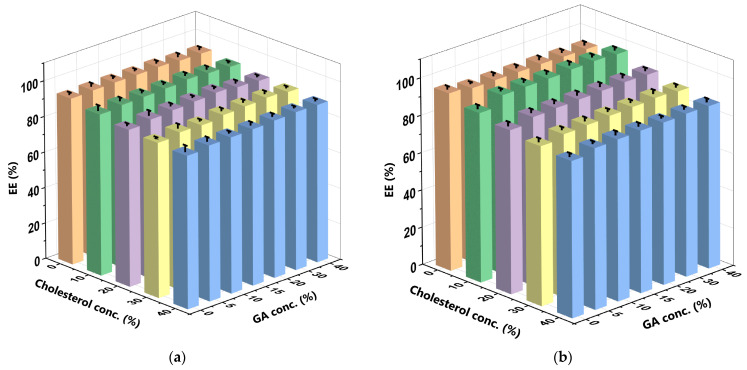
EE of the curcumin at varying C_GA_ and C_chol_ in the (**a**) SIF and (**b**) SGF. The bar graph highlights five distinct colours that represent varying C_chol_ in liposome formulations. These colours are as follows: orange for liposomes with 0% cholesterol (LC_0_), green for liposomes with 10% cholesterol (LC_10_), purple for liposomes with 20% cholesterol (LC_20_), yellow for liposomes with 30% cholesterol (LC_30_), and blue for liposomes with 40% cholesterol (LC_40_). The 10%C_chol_ + 20%C_GA_-CCL/GA formulation yielded the optimum EE (93.245% ± 1.528) for curcumin in the SIF (**a**), while the 10%C_chol_ + 30%C_GA_-CCL/GA formulation yielded the optimum EE (97.317% ± 1.261) in the SGF (**b**). The data were collected in triplicate and evaluated using ANOVA at a significance level of *p* < 0.05. Comprehensive data are available in the Supplementary Materials.

In a DDS, the EE is the percentage of the drug that is successfully encapsulated within the carrier, enabling its targeted delivery to the intended site in vivo [37,42]. Therefore, the EE of the curcumin is the amount of curcumin that had been successfully encapsulated within the CCL. The higher the amount of curcumin successfully encapsulated within the liposomes, the higher the EE of the curcumin. Figure 4 depicts the EE of the curcumin at different C_GA_ and C_chol_ in the SIF and SGF.

Cholesterol decreased the EE more significantly than GA increased it in all the CCL/GA formulations in both solutions. More specifically, when the CCL/GA formulations contained identical amounts of C_chol_ and C_GA_ and were used in the SIF, C_chol_ decreased the EE by 5%, to 8%, while the C_GA_ increased the EE by only 1%, to 5%. Similarly, when the CCL/GA formulations contained identical amounts of C_chol_ and C_GA_ and were used in the SGF, the C_chol_ decreased the EE by 7% to 12%, while the C_GA_ increased the EE by 4% to 7%, with the exception of the 0% C_chol_ variation, which decreased the EE. Hudiyanti et al. (2021) [28], similarly, found that the EE decreased when the C_chol_ increased, while Hudiyanti et al. (2022) [19] and Al Khafiz et al. (2019) [20] found that the EE increased when the C_GA_ increased. The EE of lipophilic drugs decreased in both solutions when they were encapsulated into liposomes with high C_chol_. This could be because both the molecules occupy the same space when encapsulated, specifically, between the liposome bilayers. Therefore, as the amount of space has decreased, the amount of curcumin encapsulated is lower [42]. It also indicates that the encapsulation of curcumin in the liposome membrane is anti-synergic in the presence of cholesterol and synergistic in the presence of GA as it promotes the encapsulation of curcumin. The 10%C_chol_ + 20%C_GA_-CCL/GA formulation yielded the optimum EE (93.245% ± 1.528) for curcumin in the SIF (Figure 4a), while the 10%C_chol_ + 30%C_GA_-CCL/GA formulation yielded the optimum EE (97.317% ± 1.261) in the SGF (Figure 4b). The insignificant difference in the EE in the SIF and SGF indicates that pH does not significantly alter the feasibility of encapsulating curcumin within liposomes.

### 3.3. Curcumin Loading Capacity (LC) of the CocoPLs and GA Carriers

**Figure 5:**
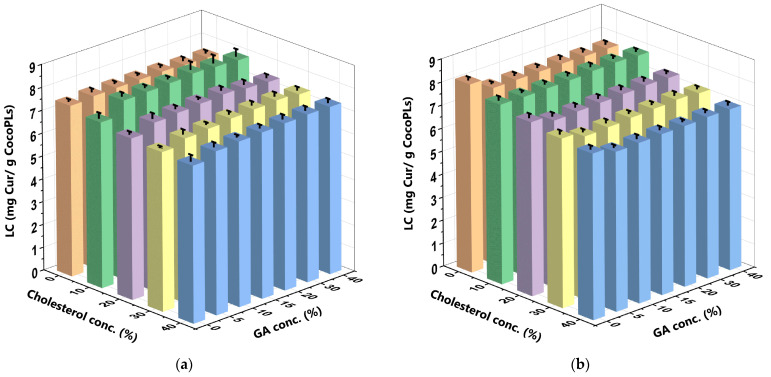
LC of the CocoPL carrier at varying C_GA_ and C_chol_ in the (**a**) SIF and (**b**) SGF. The bar graph highlights five distinct colours that represent varying C_chol_ in liposome formulations. These colours are as follows: orange for liposomes with 0% cholesterol (LC_0_), green for liposomes with 10% cholesterol (LC_10_), purple for liposomes with 20% cholesterol (LC_20_), yellow for liposomes with 30% cholesterol (LC_30_), and blue for liposomes with 40% cholesterol (LC_40_). The 10%C_chol_ + 20%C_GA_-CCL/GA formulation yielded the optimum LC of the CocoPLs (7.989 mg Cur/g CocoPLs ± 0.329) in the SIF (**a**), while the 10%C_chol_ + 30% C_GA_ CCL/GA formulation yielded the optimum LC of the CocoPLs (7.887 mg Cur/g CocoPLs ± 0.102) in the SGF (**b**). The data were collected in triplicate and evaluated using ANOVA at a significance level of *p* < 0.05. Comprehensive data are available in the Supplementary Materials.

**Figure 6:**
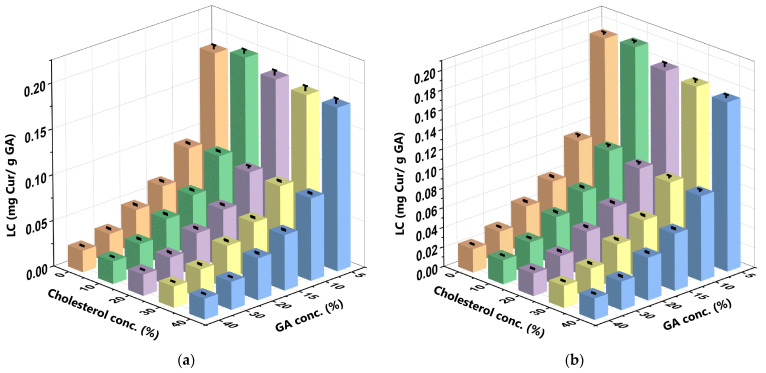
LC of the GA carrier at varying C_GA_ and C_chol_ in the (**a**) SIF and (**b**) SGF. The bar graph highlights five distinct colours that represent varying C_chol_ in liposome formulations. These colours are as follows: orange for liposomes with 0% cholesterol (LC_0_), green for liposomes with 10% cholesterol (LC_10_), purple for liposomes with 20% cholesterol (LC_20_), yellow for liposomes with 30% cholesterol (LC_30_), and blue for liposomes with 40% cholesterol (LC_40_). The 10%C_chol_ + 5%C_GA_-CCL/GA formulation yielded the optimum LC of the GA (0.196 mg Cur/g GA ± 0.004) in the SIF (**a**), while the 10%C_chol_ + 5%C_GA_-CCL/GA formulation yielded the optimum LC of the GA (0.194 mg Cur/g GA ± 0.002) in the SGF (**b**). The data were collected in triplicate and evaluated using ANOVA at a significance level of *p* < 0.05. Comprehensive data are available in the Supplementary Materials.

In a DDS, the LC is the ability of a carrier to encapsulate the drug in question. It is determined by dividing the mass of the encapsulated drug by the mass of the carrier [43]. The LC results of this present study indicate that the highest amount of curcumin that could be encapsulated within the liposomes was proportional to the mass of the carrier, specifically, CocoPLs and GA. The LC increased proportionally when the ability of the carrier to encapsulate the drug increased. Therefore, it reflects the potential and efficiency of the carrier used in the proposed DDS [44,45]. Figure 5 depicts the LC of the CocoPL carrier at different C_GA_ and C_chol_ in the SIF and SGF, while Figure 6 depicts the same for the GA carrier.

The LC results of the CocoPLs in each CCL/GA formulation in the SIF indicate that GA increases the LC significantly more than cholesterol decreases it. More specifically, when the CCL/GA formulations contained identical amounts of C_chol_ and C_GA_ and were used in the SIF, C_chol_ decreased the LC by 3%, to 8%, while the C_GA_ increased the LC by 2%, to 10%. However, when the CCL/GA formulations contained identical amounts of C_chol_ and C_GA_ and were used in the SGF, they both decreased the LC, cholesterol more so than GA. More specifically, C_chol_ decreased the LC by 9%, to 12%, while C_GA_ only decreased it by 3%, to 6%, with the exception of 10% C_chol_, which increased the LC. Much like the effect of high C_chol_ on the EE, liposomes containing high C_chol_ had limited space with which to encapsulate the curcumin [42]. The effect of cholesterol in both the SIF and SGF was similar to that of an extant study [28]. The C_GA_ affected the LC of the CocoPLs differently in each CCL/GA formulation in the SIF and SGF. More specifically, higher C_GA_ increased the LC of the CocoPLs in the SIF but decreased it in the SGF. This could be attributed to the presence of hydrolysed GA in the SGF, which decreased its LC and prevented the liposome from effectively guarding contents that are lipophilic, such as curcumin. The effect of C_GA_, particularly in the SGF, aligns with the results of a prior study [20]. The 10%C_chol_ + 20%C_GA_-CCL/GA formulation yielded the optimum LC of the CocoPLs (7.989 mg Cur/g CocoPLs ± 0.329) in the SIF (Figure 5a), while the 10%C_chol_ + 30% C_GA_ CCL/GA formulation yielded the optimum LC of the CocoPLs (7.887 mg Cur/g CocoPLs ± 0.102) in the SGF (Figure 5b). The insignificant difference in the LC in the SIF and SGF indicates that pH does not significantly affect the ability of CocoPLs to act as a carrier. The only discernible distinction lies in the variations in the formulations.

Unlike the LC of the CocoPL carrier, the GA carrier was found to lower the LC more significantly than cholesterol in both solutions. When the C_GA_ increased and the C_chol_ remained constant, the LC decreased by 0.9%. Conversely, when the C_chol_ increased and the C_GA_ remained constant, the LC decreased by 0.1%. The effect of cholesterol on the LC of GA was similar to its impact on the LC of CocoPLs in the SIF and SGF. For the GA, the decrease in the LC could be attributed to steric resistance, which disrupts the interactions required to develop a protective coating on the liposomes [46].

Furthermore, high acidity (pH < 4), such as that of the SGF, may cause the GA to hydrolyse as it is polyelectrolytic in nature [47], which, subsequently, decreases its capacity to encapsulate drugs. On the other hand, high alkalinity, such as that of the SIF, may cause the carboxylic groups in the GA to ionise, which, subsequently, creates repulsive forces between the acid groups that destabilise the structure of the GA and decrease its LC. Extant studies have reported similar effects of GA in both solutions [19,20]. The 10%C_chol_ + 5%C_GA_-CCL/GA formulation yielded the optimum LC of the GA (0.196 mg Cur/g GA ± 0.004) in the SIF (Figure 6a), while the 10%C_chol_ + 5%C_GA_-CCL/GA formulation yielded the optimum LC of the GA (0.194 mg Cur/g GA ± 0.002) in the SGF (Figure 6b). The LC of the GA was slightly higher in the SIF than in the SGF. Extant studies have, similarly, reported that the conditions in the SIF are more favourable when encapsulating curcumin with GA [19].

### 3.4. Release Rate (RR) of the Curcumin

**Figure 7:**
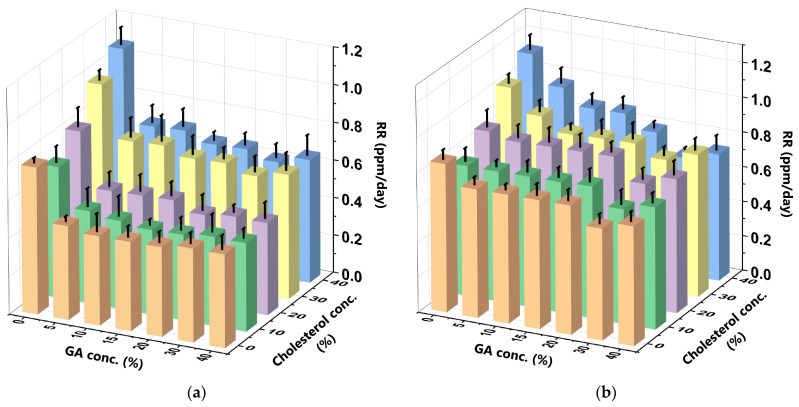
Curcumin RR at varying C_GA_ and C_chol_ in the (**a**) SIF and (**b**) SGF. The bar graph highlights five distinct colours that represent varying C_chol_ in liposome formulations. These colours are as follows: orange for liposomes with 0% cholesterol (LC_0_), green for liposomes with 10% cholesterol (LC_10_), purple for liposomes with 20% cholesterol (LC_20_), yellow for liposomes with 30% cholesterol (LC_30_), and blue for liposomes with 40% cholesterol (LC_40_). The 10%C_chol_ + 15%C_GA_-CCL/GA formulation yielded the lowest RR (0.450 ppm/day ± 0.029) in the SIF (**a**), while the 10%C_chol_ + 30%C_GA_-CCL/GA formulation yielded the lowest RR (0.657 ppm/day ± 0.082) in the SGF (**b**). The data were collected in triplicate and evaluated using ANOVA at a significance level of *p* < 0.05. Comprehensive data are available in the Supplementary Materials.

The drug RR in a DDS correlates with achieving a desired therapeutic effect at the intended site in vivo [48]. A DDS with a controlled RR releases a drug at the desired location and time while considering the therapeutic concentration and pharmacokinetic properties of the drug in question [49]. This ensures that the drug is released at the intended location without excessive dosage, thereby minimising side effects on other bodily tissues [42,50]. In this present study, the RR was determined by comparing the total amount of curcumin released over the observation period with the initial amount of curcumin in the liposome. Figure 7 depicts the curcumin RR at various C_GA_ and C_chol_ in the SIF and SGF.

The GA more significantly decreased the RR of the CCL/GA formulation in both SIF and SGF than cholesterol increased it. More specifically, when the C_GA_ increased and the C_chol_ remained constant in the SIF, the RR decreased by 25%, to 43%. However, the RR increased by 25%, to 48%, when the C_chol_ increased and the C_GA_ remained constant in the SIF. Conversely, when the C_GA_ increased and the C_chol_ remained constant in the SGF, the RR decreased by 12%, to 40%. However, the RR increased by 13%, to 32%, when the C_chol_ increased and the C_GA_ remained constant in the SGF. Therefore, GA significantly affects the RR as it is a polymer that enhances the stability of liposomes. Furthermore, the presence of GA on the outside of the liposome creates a protective layer that may improve its stability without increasing its density [26,51]. The RR results of this present study were similar to that of the EE results, wherein GA has a synergistic effect on curcumin encapsulation while cholesterol has an anti-synergistic effect. Apart from minimising the risk of liposome leakage, GA also decreases the likelihood of easily releasing the encapsulated curcumin, as its bulky structure serves as a significant steric barrier for the curcumin to traverse, thereby decreasing its RR. Furthermore, the presence of cholesterol in the same encapsulation space as curcumin increases competition for encapsulation space, which causes the curcumin to be released and increases the RR [42,52]. This finding is in line with that of extant studies [28]. The 10%C_chol_ + 15%C_GA_-CCL/GA formulation yielded the lowest RR (0.450 ppm/day ± 0.029) in the SIF (Figure 7a), while the 10%C_chol_ + 30%C_GA_-CCL/GA formulation yielded the lowest RR (0.657 ppm/day ± 0.082) in the SGF (Figure 7b). The higher curcumin RR in the SGF implies that it released curcumin faster than the SIF. This release was accelerated by the hydrolysis of GA in the acidic environment. Hudiyanti et al. (2022) reported similar findings [19].

### 3.5. Particle Size (PS), Polydispersity Index (PDI), and Zeta Potential (ζ-Potential) of the Liposomes

When developing liposome formulations for nanoparticle-scale DDSs, it is essential to consider particle size (PS) and particle size distribution, as they directly affect how they release a drug throughout the body [53]. The PS affects the surface-to-volume ratio. More specifically, smaller-sized particles have a higher surface-to-volume ratio and more surface area for nanoliposomes. Therefore, the encapsulated drug is closer to the surface and released faster. Meanwhile, larger particles have a lower surface-to-volume ratio; therefore, the encapsulated drug is released slower. The PDI, which ranges from 0 to 1, indicates the extent of heterogeneity in PS distribution in a nanoparticle system. More specifically, a PDI < 0.1 indicates a mono-dispersed PS distribution, and a PDI > 0.1 indicates a polydispersed PS distribution, while a PDI = 0–1 indicates that the PS distribution is more uniform and more homogenous, while a PDI > 1 indicates an irregular PS distribution [54,55]. Zeta (ζ)-potential was used an additional parameter to assess the stability of the nanoliposome formulations for the DDS. The ζ-potential, which is the surface charge of a nanoparticle, may indicate the stability of the colloidal system, especially in the present study, as it involves a liposomal dispersion. Particles in a colloidal system do not aggregate or flocculate when the ζ-potential is high (>30 mV or <−30 mV) due to electrostatic repulsion between the individual particles, which enhances the long-term stability of the colloidal system during storage [26,56].

Table 3 presents the PS, PDI, and ζ-potential of CCL formulations containing curcumin and varying C_chol_, while Table 4 presents the PS, PDI, and ζ-potential of the 10%C_chol-_CCL formulations containing curcumin and coated with various C_GA_.

As seen in Table 3, the PSs (>100 nm) indicate that the liposome nanoparticles are large uni-lamellar vesicles (LUVs). The present study successfully developed LUV liposomes by using the thin-layer hydration method along with sonication throughout the manufacturing process. Thin-layer hydration forms giant uni-lamellar vesicle (GUV, >1000 nm) liposomes, but sonication can be used to decrease their size to the LUV category [57,58]. As seen in Table 3, the liposome dispersion was polydispersed, and the PS distribution was fairly stable, as the PDI was 0.1–1. However, the PS distributions of the LC_20_ and LC_40_ dispersions (>0.7) fluctuated slightly. The PDI must be low, as DDSs that use lipid-based carriers, such as liposomes, require a PDI < 0.3 [54,59]. During sonication, the vibration intensity and the sonication duration should be evaluated. As the ζ-potential was <−30 mV, the electrostatic stability of the liposome dispersion in all the formulations was excellent. Therefore, higher C_chol_ enhances the electrostatic stability of a liposome by increasing the negative charge of its surfaces, which ensures its long-term stability by effectively minimising the risk of flocculation during storage [56,60]. The ζ-potential of the LC_0_ dispersion was negative due to the ionisation of the phosphate groups present in the phospholipids, which negatively charged the membrane of the liposome [61]. As the ζ-potentials of the LC_10_ (−34.6 mV) and LC_20_ (−31.6 mV) dispersions were more negative than that of the LC_0_ (−30.9 mV) dispersion, C_chol_ affects the ζ-potentials of the liposomes. The ζ-potentials of the liposomes in this present study were significantly negative due to interactions between the cholesterol and the phospholipids. The hydroxyl group of cholesterol can create hydrogen (H) bonds with the phosphate group of phospholipids [62]. The hydroxyl group of cholesterol also tends to form H bonds with the choline group of the phosphatidylcholines. Therefore, the cholesterol causes interactions to occur between the hydroxyl and choline groups, which produces liposomes with significantly negatively charged surfaces. More specifically, the positively charged choline groups are drawn to the membrane, while the negatively charged hydroxyl groups are drawn to the surface of the membrane [61,63].

As seen in Table 4, the PSs of the liposome dispersions of each formulation indicate that the liposome nanoparticles are LUVs, and the PDI indicates that the liposome dispersion is polydispersed. Therefore, its homogeneity must be increased to achieve a more stable PS distribution. Meanwhile, the ζ-potentials indicate that the electrostatic stability of the LC_10_ formulation when combined with the GA matrix was poor (>−30 mV). Therefore, the long-term stability of formulations that have been coated with C_GA_ is poor, and they are prone to flocculation during storage, unlike the formulation sans C_GA_ (LC_10_G_0_). Nevertheless, the ζ-potentials of the LC_10_G_10_ (−20.6 mV) and LC_10_G_20_ (−19.9 mV) dispersions were better than that of the LC_10_G_0_ (−34.6 mV) dispersion. Therefore, the GA matrix affects the ζ-potential of the liposomes. Adding GA to the liposome formulation forms a thin protective layer as it can interact with the outer surface, or external lipid bilayer, of the liposomes. However, higher levels of C_GA_ alter both the morphology and surface charge of liposomes, which increases the likelihood of liposome aggregation and decreases its short-term stability [26,64].

### 3.6. Antioxidant Activity (IR) of the Curcumin

**Figure 8:**
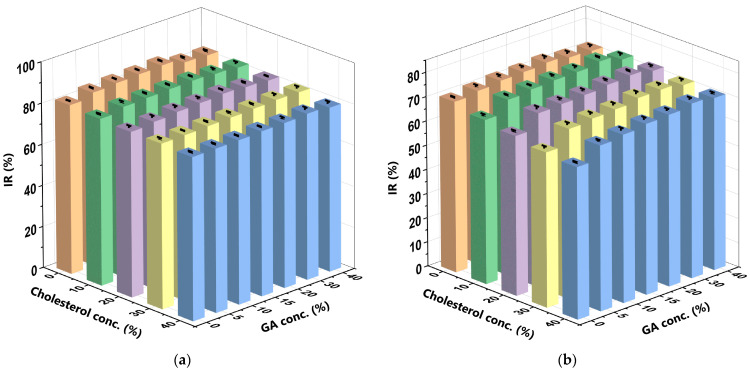
IR of curcumin at various C_GA_ and C_chol_ in the (**a**) SIF and (**b**) SGF. The bar graph highlights five distinct colours that represent varying C_chol_ in liposome formulations. These colours are as follows: orange for liposomes with 0% cholesterol (LC_0_), green for liposomes with 10% cholesterol (LC_10_), purple for liposomes with 20% cholesterol (LC_20_), yellow for liposomes with 30% cholesterol (LC_30_), and blue for liposomes with 40% cholesterol (LC_40_). The 10%C_chol_ + 20%C_GA_-CCL/GA formulation yielded the optimum IR (84.081% ± 0.297) in the SIF (**a**), while the 10%C_chol_ + 30%C_GA_-CCL/GA formulation yielded the optimum IR (75.439% ± 0.356) in the SGF (**b**). The data were collected in triplicate and evaluated using ANOVA at a significance level of *p* < 0.05. Comprehensive data are available in the Supplementary Materials.

The IR indicates the potential of an antioxidant compound, such as curcumin, to scavenge free radicals, inhibit the formation of reactive oxygen species (ROS), and prevent the oxidation of nutrients, especially lipids and proteins [65]. This present study used a DPPH scavenging activity assay to determine the IR of the curcumin encapsulated in the CCL/GA formulations [66,67]. DPPH is a stable free radical that exhibits its maximum absorption in ethanol at λ = 515–517 nm and appears dark purple in colour. When the H donor atoms of the antioxidant compound capture free radicals in the DPPH, it turns colourless or pale yellow [68,69]. The ability of curcumin to scavenge DPPH is proportional to the IR measured. Curcumin is a potent antioxidant as it can effectively scavenge DPPH free radicals, even at low C_cur_ [28,70]. Figure 8 depicts the IR of curcumin at various C_GA_ and C_chol_ in the SIF and SGF.

The results of the CCL/GA formulation in the SIF indicate that cholesterol more significantly decreases IR than GA increases it. When the C_chol_ increased and the C_GA_ remained constant, the IR decreased by 4%, to 7%, but it increased by only 2%, to 5%, when the C_GA_ increased and the C_chol_ remained constant. Conversely, GA more significantly increases IR in the SGF than cholesterol decreases it. When the C_GA_ increased and the C_chol_ remained constant, the IR increased by 4%, to 15%, but it decreased by only 1% to 11% when the C_chol_ increased and the C_GA_ remained constant. Extant studies have reported similar effects of GA [19] and cholesterol [28] in SIF and SGF. As GA is a carrier and an antioxidant, it improves the IR of curcumin in direct proportion to the amount of GA used [71,72]. The 10%C_chol_ + 20%C_GA_-CCL/GA formulation yielded the optimum IR (84.081% ± 0.297) in the SIF (Figure 8a), while the 10%C_chol_ + 30%C_GA_-CCL/GA formulation yielded the optimum IR (75.439% ± 0.356) in the SGF (Figure 8b). The IR of curcumin was higher in the SIF than in the SGF due to the inherent properties of curcumin as it undergoes degradation more quickly in alkaline environments than in neutral and acidic environments. Therefore, the amount of non-degradable curcumin that reacts with the DPPH free radicals will differ. Curcumin exists as an enolate in the heptadienone chain (electron donor) in alkaline environments but in a protonated form (H donor) in neutral and acidic environments. It also exhibits low solubility in acidic environments. However, curcumin can only interact with DPPH free radical in its H donor form; therefore, curcumin has higher IR in the SIF [19,28].

Figure 8 depicts the IR as the antioxidant activity dominated by curcumin. Table 5 depicts the IR of the CocoPLs + cholesterol carrier sans curcumin, while Table 6 depicts that of the GA carrier sans curcumin.

As seen in Table 5, the highest IR of the CocoPLs + C_chol_ carriers sans curcumin was 3.549% in the SIF and 1.869% in the SGF. Meanwhile, that of the C_GA_ carrier sans curcumin was 6.026% in the SIF and 4.896% in the SGF (Table 6). The IR of curcumin only was 85.311% in the SIF and 86.94% in the SGF. Therefore, the IR of both the CocoPLs and C_GA_ carriers was significantly lower than that of curcumin (IR of curcumin in SIF ≤ 7% and in SGF ≤ 6%). As such, the IR when CocoPLs or C_GA_ are used to encapsulate curcumin is the IR of the curcumin alone. Therefore, the IR of the CocoPLs and C_GA_ carriers can be overlooked.

## 4. Conclusions

The present study successfully encapsulated curcumin in a liposome-based DDS comprising phospholipids, cholesterol, and GA polymer in SIF (neutral to slightly alkaline) and SGF (acidic). The liposomes were nanoparticles with comparable uniform PS distribution. The FTIR spectra of the CCL/GA formulation indicate the absence of new characteristic peaks that differ from those of its constituent components. Therefore, the interactions that occurred when encapsulating curcumin into the CCL/GA matrix were non-covalent. Increasing the C_chol_ decreased the EE, LC, and IR but increased the RR. Meanwhile, increasing the C_GA_ increased both the EE and IR but decreased the LC and RR. These outcomes were observed in both the SIF and SGF. The IR of curcumin was higher in the SIF, but its RR was faster in the SGF. The 10%C_chol_ + 20%C_GA_ CCL/GA formulation was the best for SIF as its EE = 93.245%, LC of CocoPLs = 7.989 mg Cur/g CocoPLs, LC of GA = 0.196 mg Cur/g GA (10%C_chol_ + 5%C_GA_), IR = 84.081%, and RR = 0.450 ppm/day (10%C_chol_ + 15%C_GA_). The 10%C_chol_ + 30%C_GA_ CCL/GA formulation was the best for SGF as its EE = 97.317%, LC of CocoPLs = 7.887 mg Cur/g CocoPLs, LC of GA = 0.194 mg Cur/g GA (10%C_chol_ + 5%C_GA_), IR = 75.439%, and RR = 0.657 ppm/day. The CCL/GA formulations performed better in the SIF than in the SGF. As this present study only used PBS as a simulation solution, future studies should examine using bile salt in the simulation solution. Nevertheless, the findings of this present study may serve as a guideline for formulating liposomal curcumin formulations, especially for the gastrointestinal tract.

## Figures and Tables

**Table 1:** Curcumin encapsulated within cocoliposome (CCL) formulations.

CCL Formulation	Composition (*w*/*w*/*w*; mg)		
	CocoPLs	Cholesterol p.a. (Chol)	* Curcumin p.a. (Cur)
LC_0_	125	0	1
LC_10_	125	12.5	1
LC_20_	125	25	1
LC_30_	125	37.5	1
LC_40_	125	50	1

* Curcumin p.a. in this table will be referred to as C_0_ curcumin or the initial curcumin p.a. concentration.

**Table 2:** Curcumin encapsulated within cocoliposomes with GA matrix coating (CCL/GA) formulations.

CCL Formulation	C_GA_						
	0%	5%	10%	15%	20%	30%	40%
LC_0_	^1^ LC_0_G_0_	^3^ LC_0_G_5_	LC_0_G_10_	LC_0_G_15_	LC_0_G_20_	LC_0_G_30_	LC_0_G_40_
LC_10_	LC_10_G_0_	LC_10_G_5_	LC_10_G_10_	LC_10_G_15_	LC_10_G_20_	LC_10_G_30_	LC_10_G_40_
LC_20_	LC_20_G_0_	LC_20_G_5_	LC_20_G_10_	LC_20_G_15_	LC_20_G_20_	LC_20_G_30_	LC_20_G_40_
LC_30_	LC_30_G_0_	LC_30_G_5_	LC_30_G_10_	LC_30_G_15_	LC_30_G_20_	LC_30_G_30_	LC_30_G_40_
LC_40_	LC_40_G_0_	LC_40_G_5_	LC_40_G_10_	LC_40_G_15_	LC_40_G_20_	LC_40_G_30_	^2^ LC_40_G_40_

^1^ LC_0_G_0_ means that the CCL formulation contained 0% C_chol_ and was coated with a 0% C_GA_. ^2^ LC_40_G_40_ means that the CCL formulation contained 40% C_chol_ and was coated with 40% C_GA_. The codes from the other formulations can be adjusted to match the provided examples. ^3^ To produce the LC_0_G_5_ formulation, 6.3 mg GA (5%) was required based on the total mass of the LC_0_ composition (125 mg CocoPLs + 0 mg cholesterol p.a. + 1 mg curcumin p.a.) in the CCL formulation (Table 1).

**Table 3:** PS, PDI, and ζ-potential of CCL containing curcumin and various C_chol_ in the SIF.

	LC_0_	LC_10_	LC_20_	LC_30_	LC_40_
PS (nm)	305.6	704.3	366.8	272.9	602.9
PDI	0.546	0.588	0.847	0.464	0.720
ζ-potential (mV)	−30.9	−34.6	−31.6	−31.2	−34.1

**Table 4:** PS, PDI, and ζ-potential of 10%C_chol_-CCL formulations containing curcumin and coated with various C_GA_ in the SIF.

	LC_10_G_0_	LC_10_G_5_	LC_10_G_10_	LC_10_G_15_	LC_10_G_20_	LC_10_G_30_	LC_10_G_40_
PS (nm)	704.3	838.1	991.4	591.4	606.9	852.5	898.4
PDI	0.588	0.574	0.640	0.647	0.494	0.404	0.571
ζ-potential (mV)	−34.6	−20.6	−19.9	−14.4	−12.7	−9.1	−7.4

**Table 5:** IR of the CocoPLs + C_chol_ carrier sans curcumin.

	LC_0_	LC_10_	LC_20_	LC_30_	LC_40_
SIF	2.788%	3.042%	3.169%	3.549%	3.549%
SGF	0.932%	1.242%	1.398%	1.863%	1.863%

**Table 6:** IR of the C_GA_ carrier sans curcumin.

	G_5_	G_10_	G_15_	G_20_	G_30_
SIF	1.695%	2.245%	3.013%	3.766%	4.520%
SGF	1.305%	1.794%	2.447%	2.936%	3.589%

## Data Availability

All data generated or analysed during this study are included in this published article.

## Supplementary Materials

The following supporting information can be downloaded at https://www.mdpi.com/article/10.3390/polym16070944/s1, Table S1: Tabulation of analysis results from CCL/GA formulations in SIF solution. Table S2: Tabulation of analysis results from CCL/GA formulations in SGF solution.
