# Supplementary material for: Prospect of Gum Arabic–Cocoliposome Matrix to Encapsulate Curcumin for Oral Administration
Source: Polymers (Basel). 2024 Mar 29;16(7):944. doi: 10.3390/polym16070944 (PMC11013629; doi:10.3390/polym16070944)
Supplement: Supplementary file 1 [file polymers-16-00944-s001.zip › polymers-2865557-supplementary.pdf]

Table S1. Tabulation of analysis results from CCL/GA formulations in SIF solution

CCL/GA Formulation	EE (%)	LC CocoPLs (mg Cur/g CocoPLs)	LC GA (mg Cur/g GA)	IR (%)	RR (ppm/day)
LC_0G_0	93.373 ± 1.144	7.471 ± 0.092	-	82.129 ± 0.125	0.778 ± 0.025
$LC_{10}G_0$	90.474 ± 2.860	7.239 ± 0.229	-	80.989 ± 0.453	0.696 ± 0.084
$LC_{20}G_0$	88.047 ± 1.239	7.045 ± 0.099	-	80.228 ± 0.796	0.801 ± 0.100
$LC_{30}G_0$	86.901 ± 0.762	6.953 ± 0.061	-	80.101 ± 0.638	0.966 ± 0.051
$LC_{40}G_0$	85.822 ± 3.336	6.867 ± 0.267	-	79.214 ± 0.492	1.073 ± 0.090
LC_0G_5	93.812 ± 1.755	7.552 ± 0.141	0.189 ± 0.004	84.371 ± 0.354	0.497 ± 0.025
$LC_{10}G_5$	91.608 ± 1.872	7.849 ± 0.160	0.196 ± 0.004	82.923 ± 0.649	0.491 ± 0.095
$LC_{20}G_5$	89.581 ± 2.630	7.385 ± 0.217	0.185 ± 0.005	81.222 ± 0.925	0.513 ± 0.074
$LC_{30}G_5$	87.631 ± 2.291	7.196 ± 0.188	0.180 ± 0.005	80.314 ± 0.169	0.692 ± 0.100
$LC_{40}G_5$	86.410 ± 2.225	7.142 ± 0.184	0.179 ± 0.005	79.356 ± 0.438	0.687 ± 0.089
LC_0G_{10}	94.013 ± 1.132	7.568 ± 0.091	0.095 ± 0.001	85.104 ± 0.161	0.473 ± 0.093
$LC_{10}G_{10}$	91.923 ± 1.235	7.876 ± 0.106	0.098 ± 0.001	83.357 ± 0.471	0.469 ± 0.105
$LC_{20}G_{10}$	90.497 ± 1.482	7.461 ± 0.122	0.093 ± 0.002	82.116 ± 0.826	0.518 ± 0.084
$LC_{30}G_{10}$	88.485 ± 0.988	7.266 ± 0.081	0.091 ± 0.001	80.742 ± 0.381	0.699 ± 0.135
$LC_{40}G_{10}$	87.258 ± 1.058	7.212 ± 0.087	0.090 ± 0.001	79.502 ± 0.293	0.694 ± 0.091
LC_0G_{15}	94.080 ± 1.068	7.574 ± 0.086	0.063 ± 0.001	85.592 ± 0.193	0.473 ± 0.056
$LC_{10}G_{15}$	92.363 ± 1.153	7.914 ± 0.099	0.066 ± 0.001	83.792 ± 0.364	0.450 ± 0.029
$LC_{20}G_{15}$	91.151 ± 0.928	7.514 ± 0.077	0.063 ± 0.001	82.563 ± 0.721	0.522 ± 0.071
$LC_{30}G_{15}$	89.667 ± 1.462	7.363 ± 0.120	0.061 ± 0.001	81.027 ± 0.592	0.657 ± 0.096
$LC_{40}G_{15}$	87.780 ± 1.726	7.256 ± 0.143	0.060 ± 0.001	79.941 ± 0.278	0.648 ± 0.043
LC_0G_{20}	94.281 ± 1.267	7.590 ± 0.102	0.047 ± 0.001	86.081 ± 0.369	0.474 ± 0.056
$LC_{10}G_{20}$	93.245 ± 1.528	7.989 ± 0.329	0.050 ± 0.001	84.081 ± 0.297	0.454 ± 0.065
$LC_{20}G_{20}$	91.871 ± 0.989	7.574 ± 0.151	0.047 ± 0.001	83.159 ± 0.185	0.473 ± 0.082
$LC_{30}G_{20}$	90.258 ± 1.382	7.412 ± 0.156	0.046 ± 0.001	81.455 ± 0.478	0.662 ± 0.059
$LC_{40}G_{20}$	88.368 ± 1.617	7.304 ± 0.187	0.046 ± 0.001	80.088 ± 0.659	0.652 ± 0.061
LC_0G_{30}	93.678 ± 1.825	7.541 ± 0.147	0.031 ± 0.001	83.761 ± 0.583	0.496 ± 0.057
$LC_{10}G_{30}$	92.615 ± 1.617	7.935 ± 0.329	0.033 ± 0.001	83.502 ± 0.679	0.472 ± 0.094
$LC_{20}G_{30}$	90.759 ± 1.539	7.482 ± 0.177	0.031 ± 0.001	82.563 ± 0.526	0.492 ± 0.037
$LC_{30}G_{30}$	90.849 ± 1.481	7.461 ± 0.162	0.031 ± 0.001	81.883 ± 0.814	0.622 ± 0.081
$LC_{40}G_{30}$	88.824 ± 1.014	7.342 ± 0.158	0.031 ± 0.0003	80.673 ± 0.957	0.612 ± 0.073
LC_0G_{40}	93.544 ± 1.452	7.531 ± 0.117	0.024 ± 0.0004	83.394 ± 0.539	0.495 ± 0.073
$LC_{10}G_{40}$	91.986 ± 0.862	7.881 ± 0.309	0.025 ± 0.0002	82.779 ± 0.826	0.469 ± 0.049
$LC_{20}G_{40}$	90.104 ± 0.951	7.428 ± 0.147	0.023 ± 0.0002	81.967 ± 0.271	0.489 ± 0.083
$LC_{30}G_{40}$	89.864 ± 0.924	7.380 ± 0.132	0.023 ± 0.0002	81.740 ± 0.859	0.659 ± 0.091
$LC_{40}G_{40}$	88.629 ± 1.128	7.326 ± 0.162	0.023 ± 0.0003	79.795 ± 0.937	0.654 ± 0.105

Table S2. Tabulation of analysis results from CCL/GA formulations in SGF solution

CCL/GA Formulation	EE (%)	LC CocoPLs (mg Cur/g CocoPLs)	LC GA (mg Cur/g GA)	IR (%)	RR (ppm/day)
LC_0G_0	95.086 ± 1.460	8.173 ± 0.125	-	70.497 ± 0.295	0.851 ± 0.053
$LC_{10}G_0$	90.630 ± 1.972	7.790 ± 0.169	-	67.702 ± 0.573	0.749 ± 0.074
$LC_{20}G_0$	87.617 ± 2.054	7.531 ± 0.177	-	65.839 ± 0.489	0.856 ± 0.096
$LC_{30}G_0$	85.232 ± 1.834	7.326 ± 0.158	-	63.820 ± 0.724	1.017 ± 0.051
$LC_{40}G_0$	83.537 ± 1.738	7.180 ± 0.149	-	62.578 ± 0.358	1.122 ± 0.083
LC_0G_5	93.657 ± 0.951	7.671 ± 0.078	0.192 ± 0.002	71.701 ± 0.657	0.738 ± 0.042
$LC_{10}G_5$	95.920 ± 1.145	7.773 ± 0.093	0.194 ± 0.002	72.727 ± 0.384	0.747 ± 0.036
$LC_{20}G_5$	90.372 ± 1.853	7.256 ± 0.149	0.181 ± 0.004	71.889 ± 0.975	0.824 ± 0.071
$LC_{30}G_5$	87.324 ± 1.376	7.072 ± 0.111	0.177 ± 0.003	70.156 ± 0.783	0.884 ± 0.064
$LC_{40}G_5$	86.004 ± 1.269	6.905 ± 0.102	0.173 ± 0.003	68.095 ± 0.517	0.959 ± 0.085
LC_0G_{10}	93.789 ± 1.478	7.682 ± 0.121	0.096 ± 0.002	72.655 ± 0.579	0.739 ± 0.031
$LC_{10}G_{10}$	96.186 ± 1.516	7.795 ± 0.123	0.097 ± 0.002	73.684 ± 0.176	0.750 ± 0.063
$LC_{20}G_{10}$	90.909 ± 1.823	7.299 ± 0.146	0.091 ± 0.002	72.350 ± 0.634	0.829 ± 0.082
$LC_{30}G_{10}$	87.857 ± 1.792	7.115 ± 0.145	0.089 ± 0.002	71.094 ± 0.714	0.809 ± 0.035
$LC_{40}G_{10}$	86.407 ± 2.015	6.937 ± 0.162	0.087 ± 0.002	69.206 ± 0.932	0.867 ± 0.059
LC_0G_{15}	93.855 ± 1.355	7.687 ± 0.111	0.064 ± 0.001	72.973 ± 0.668	0.739 ± 0.065
$LC_{10}G_{15}$	96.319 ± 1.678	7.806 ± 0.136	0.065 ± 0.001	74.163 ± 0.561	0.751 ± 0.055
$LC_{20}G_{15}$	91.245 ± 1.954	7.326 ± 0.157	0.061 ± 0.001	72.811 ± 0.628	0.832 ± 0.072
$LC_{30}G_{15}$	88.590 ± 1.295	7.174 ± 0.105	0.060 ± 0.001	71.563 ± 0.851	0.815 ± 0.081
$LC_{40}G_{15}$	86.743 ± 1.776	6.964 ± 0.143	0.058 ± 0.001	70.317 ± 0.882	0.871 ± 0.068
LC_0G_{20}	93.987 ± 1.787	7.698 ± 0.146	0.048 ± 0.001	73.609 ± 0.888	0.740 ± 0.062
$LC_{10}G_{20}$	97.051 ± 1.851	7.865 ± 0.150	0.049 ± 0.001	74.322 ± 0.768	0.756 ± 0.072
$LC_{20}G_{20}$	91.648 ± 1.923	7.358 ± 0.154	0.046 ± 0.001	73.733 ± 0.524	0.836 ± 0.055
$LC_{30}G_{20}$	89.056 ± 1.358	7.212 ± 0.110	0.045 ± 0.001	72.969 ± 0.919	0.820 ± 0.091
$LC_{40}G_{20}$	86.810 ± 1.112	6.970 ± 0.089	0.044 ± 0.001	70.794 ± 0.667	0.792 ± 0.036
LC_0G_{30}	93.723 ± 1.455	7.676 ± 0.119	0.032 ± 0.0005	72.496 ± 0.767	0.640 ± 0.055
$LC_{10}G_{30}$	97.317 ± 1.261	7.887 ± 0.102	0.033 ± 0.0004	75.439 ± 0.356	0.657 ± 0.082
$LC_{20}G_{30}$	92.051 ± 1.763	7.391 ± 0.142	0.031 ± 0.001	74.347 ± 0.415	0.711 ± 0.061
$LC_{30}G_{30}$	89.656 ± 1.952	7.261 ± 0.158	0.030 ± 0.001	73.125 ± 0.777	0.756 ± 0.048
$LC_{40}G_{30}$	87.281 ± 1.544	7.008 ± 0.124	0.029 ± 0.001	72.063 ± 0.695	0.674 ± 0.036
LC_0G_{40}	93.592 ± 1.752	7.666 ± 0.143	0.024 ± 0.0004	72.019 ± 0.771	0.684 ± 0.055
$LC_{10}G_{40}$	96.785 ± 1.551	7.843 ± 0.126	0.025 ± 0.0004	73.046 ± 0.852	0.700 ± 0.054
$LC_{20}G_{40}$	91.783 ± 1.365	7.369 ± 0.110	0.023 ± 0.0003	72.965 ± 0.648	0.768 ± 0.078
$LC_{30}G_{40}$	88.856 ± 1.446	7.196 ± 0.117	0.022 ± 0.0004	71.759 ± 0.759	0.818 ± 0.066
$LC_{40}G_{40}$	87.079 ± 1.761	6.991 ± 0.141	0.022 ± 0.0004	71.111 ± 0.558	0.728 ± 0.081
